# Early Evaluation and the Effect of Socioeconomic Factors on Neurodevelopment in Infants with Tetralogy of Fallot

**DOI:** 10.1007/s00246-020-02525-6

**Published:** 2021-02-03

**Authors:** Emmanuelle Favilla, Jennifer A. Faerber, Lyla E. Hampton, Vicky Tam, Grace DeCost, Chitra Ravishankar, J. William Gaynor, Alisa Burnham, Daniel J. Licht, Laura Mercer-Rosa

**Affiliations:** 1 Division of Cardiology, Children’s Hospital of Philadelphia, Philadelphia, PA 19104 USA; 2 Division of Neurology, Children’s Hospital of Philadelphia, Philadelphia, PA USA; 3 Department of Pediatrics, Children’s Hospital of Philadelphia, Philadelphia, PA USA; 4 Department of Biomedical and Health Informatics, Data Science and Biostatistics Unit, Children’s Hospital of Philadelphia, Philadelphia, PA USA; 5 Department of Child & Adolescent Psychiatry and Behavioral Sciences, Children’s Hospital of Philadelphia, Philadelphia, PA USA; 6 Cartographic Modeling Lab, University of Pennsylvania, Philadelphia, PA USA; 7 Division of Cardiothoracic Surgery, Children’s Hospital of Philadelphia, Philadelphia, PA USA

**Keywords:** Tetralogy of fallot, Neurodevelopment, Socioeconomic status

## Abstract

Neurodevelopmental sequelae are prevalent among patients with congenital heart defects (CHD). In a study of infants and children with repaired tetralogy of Fallot (TOF), we sought to identify those at risk for abnormal neurodevelopment and to test associations between socioeconomic and medical factors with neurodevelopment deficits. Single-center retrospective observational study of patients with repaired TOF that were evaluated at the institution’s Cardiac Kids Developmental Follow-up Program (CKDP) between 2012 and 2018. Main outcomes included neurodevelopmental test scores from the Bayley Infant Neurodevelopmental Screener (BINS), Peabody Developmental Motor Scale (PDMS), and Bayley Scales of Infant and Toddler Development, Third Edition (Bayley-III). Mixed effects linear regression and marginal logistic regression models tested relationships between patient characteristics and outcomes. Sub-analyses were conducted to test correlations between initial and later neurodevelopment tests. In total, 49 patients were included, predominantly male (*n* = 33) and white (*n* = 28), first evaluated at a median age of 4.5 months. Forty-three percent of patients (*n* = 16) had deficits in the BINS, the earliest screening test. Several socioeconomic parameters and measures of disease complexity were associated with neurodevelopment, independently of genetic syndrome. Early BINS and PDMS performed in infancy were associated with Bayley-III scores performed after 1 year of age. Early screening identifies TOF patients at risk for abnormal neurodevelopment. Socioeconomic factors and disease complexity are associated with abnormal neurodevelopment and should be taken into account in the risk stratification and follow-up of these patients. Early evaluation with BINS and PDMS is suggested for detection of early deficits.

**Supplementary Information:**

The online version contains supplementary material available at 10.1007/s00246-020-02525-6.

## Introduction

Tetralogy of Fallot (TOF) is the most common cyanotic congenital heart defect (CHD), with a yearly incidence of one per 2500 live births in the United States [1]. Advances in surgical and medical care have led to excellent survival of patients with TOF into adulthood, but long-term morbidities have emerged, of which neurodevelopmental deficits and impaired functional status are prevalent and can be debilitating [2–4]. Patient factors, management strategies, and disease course are known to impact these outcomes, which have been extensively studied in other severe CHD groups, including hypoplastic left heart syndrome and transposition of the great arteries, primarily using the Bayley Scale for Infant and Toddler Development, Third edition (Bayley-III) and the Wechsler Preschool and Primary Scale of Intelligence in children and adolescents [2, 5–10]. The Bayley Infant Neurodevelopmental Screener (BINS) has been used to detect early deficits in high-risk neonatal groups, including premature infants and those with other congenital anomalies, allowing for risk stratification and intervention. To our knowledge, these early tests have not been studied in patients with congenital heart disease, in particular in those with TOF [11, 12]. Further, the association of social determinants of health with overall outcomes in children is well known, but the association between socioeconomic factors and neurodevelopment status has not been extensively studied in patients with TOF [13–15].

In this single-center retrospective cohort study, we set out to conduct a twofold preliminary analysis of neurodevelopment in infants and children with repaired TOF. First, we described the neurodevelopmental status in this group and identified socioeconomic, patient, and medical management factors associated with neurodevelopment and with referral for early intervention (EI) therapy. Next, we conducted sub-analyses to test the association between the screening tests administered in infancy (BINS and PDMS) with the Bayley-III test, which is a more definitive test of neurodevelopment performed after one year of age.

## Methods

### Study Design and Data Source

We conducted a retrospective analysis of survivors of TOF repair who participated in a single-center prospective cohort study (Outcome in TOF) [16].

### Study Population

This analysis included patients from Outcome in TOF who also underwent clinically based neurodevelopmental assessment at the Children’s Hospital of Philadelphia Cardiac Kids Developmental Follow-up Program (CKDP) between 2012 and 2018. Inclusion criteria for this analysis were (1) TOF repair between 0 and 12 months of age and (2) neurodevelopment evaluation performed between 0 and 4 years of age at the CKDP. Patients with genetic syndromes were included.

Patient data were available from the research database (REDCap®) and included demographic, pre-operative, operative, and post-operative factors. Socioeconomic data were obtained from the patient’s census block group as a proxy for the neighborhood based on the geocoded home address at the time of the first CKDP evaluation. The 2018 American Community Survey (ACS) is an ongoing nationwide survey performed by the U.S. Census Bureau that gathers demographic and socioeconomic characteristics with geospatial data. The ACS was used to calculate a social disorganization index in block group level. This index reflects multiple socioeconomic status indicators of disorganization, calculated as the sum of the characteristics outlined in Table 1 [17, 18]. Other neighborhood-level factors included income, unemployment, tenure, and education.

**Table 1:** Description of the patient population

Demographics	Study cohort (*n* = 49)	
	N	Percentage
Male	33	67%
Race		
Non-hispanic caucasian	28	57%
Hispanic	10	20%
Non-hispanic Black	8	16%
Non-hispanic other	2	4%
Primary caregiver education		
Less than 12 years	1	2%
13–15 years	18	37%
Greater than 16 years	12	24%
Unknown	18	37%

Neighborhood socioeconomic factors	Median	Interquartile range
Social disadvantage index (Z-Score)	− 0.37	(− 0.97, 0.17)
Median age	36.85	(33.8, 41.65)
Median household income	69,703	(51,731, 86,807)
Non-hispanic African American	6%	(1%, 23%)
Households receiving public assistance	2%	(0%, 5%)
Female-headed households with children under 18	8%	(3%, 12%)
Hispanic	7%	(3%, 15%)
High school dropouts	8%	(6%, 16%)
Householders who moved into unit within the last 3 years	4%	(3%, 13%)
Without a vehicle	4%	(2%, 13%)
Households with > 1 person per room	0%	(0%, 3%)
Renter-occupied houses	27%	(18%, 49%)
Overall unemployment	5%	(3%, 9%)
Vacant	10%	(5%, 14%)
Non-hispanic white	74%	(45%, 86%)
Population density (per square mile)	3251.38	(1070.82, 10760.49)
Low-income persons (100–149% poverty level)	4%	(1%, 11%)
Low-income persons (< 100% poverty level)	9%	(4%, 20%)

Medical history	Median	Interquartile Range
Gestational age (weeks)	38	(37,39)
Birth weight (kg)	3.03	(2.58, 3.41)
Head circumference at birth (percentile)	18	(8,31)
	N	Percentage
Extra cardiac Malformation	31	63%
Genetics diagnosis	20	41%
22q11 deletion	5	10%
Trisomy 21	6	12%
Cardiac anatomy		
Pulmonary valve anatomy		
Stenosis	39	80%
Atresia/ Absent valve leaflets	10	20%
TOF atrioventricular canal	2	4%
Continuous pulmonary arteries	46	94%
AP collaterals	7	14%
Right aortic arch	16	33%

Surgical history	N	Percentage
Palliative surgery/intervention	3	7%
Neonatal repair	10	20%
	Median	Interquartile Range
Age at TOF repair operation (days)	90	(20, 133)
Oxygen Saturation at the time of surgery (%)	92	(81.5, 96.5)
Weight at the time of surgery (kg)	3.4	(2.6, 4.38)
BSA at the time of surgery (m2)	0.22	(0.19, 0.26)
Intra-operative		
Number of cardiopulmonary bypass runs	1	(1,1)
Total cardiopulmonary bypass time (in minutes)	67	(39, 93)

Post-operative complications	N	Percentage
Catheterization	12	24%
Arrhythmia requiring treatment	11	22%
Extra-corporal membrane oxygenation	4	8%
Pleural effusion requiring chest tube	4	8%
Cardiac arrest and cardiopulmonary resuscitation	3	6%
Pneumothorax requiring drainage	2	4%
Pericardial effusion requiring pericardiocentesis	1	2%
Seizure requiring treatment	1	2%
Post-operative length of stay (days), Median (Interquartile Range)	7.5	(6,12)

Patient and clinical factors (exposures) included pre-operative (birth history, cardiac anatomy, pre-operative use of prostaglandin, palliative procedures prior to TOF repair), operative, and post-operative (cardiopulmonary bypass (CPB) duration, number of CPB runs, lowest pH and temperature during CPB, post-operative complications, and length of hospital stay) factors. Genetic syndrome was categorized as follows: 1) 22q11 0.2 deletion syndrome and/or trisomy 21, 2) other genetic syndrome, and 3) no identified genetic syndrome.

### Assessment of Neurodevelopment (Outcome)

Data from clinical neurodevelopmental tests performed as an outpatient in the CKDP were retrospectively collected. Patients were eligible for evaluation in the CKDP if they had either a catheter-based or surgical intervention within the first six months of life, and those with a neonatal hospitalization longer than ten days. The CKDP team is composed of a pediatrician, a clinical psychologist, and occupational and physical therapists. Visits included parental interviews, standardized assessment, and feedback to parents with recommendations for intervention and follow-up on a yearly basis at a minimum, regardless of the presence or lack of deficits. The following standardized measures were performed based on age at testing: Bayley Infant Neurodevelopmental Screener (BINS), Peabody Developmental Motor Scale (PDMS) [19], and Bayley Scales of Infant and Toddler Development Third Edition (Bayley-III) [20]. Domains assessed by BINS and Bayley-III include expressive and receptive language, cognition, fine, and gross motor skills; the PDMS assesses fine and gross motor skills. The BINS is administered during the first year of life, and scored as “normal,” “emerging,” or “at risk” development. The normative mean (standard deviation) for both Bayley-III and PDMS is 100 ± 15. The PDMS test is performed during the first year of life as well, while the Bayley-III is performed after 1 year of age (Table S1). Referral to early intervention was chosen as one of the outcomes of interest since a referral could be prompted by caregiver and/or provider concerns independently of test results. If there were multiple administrations of the BINS, Bayley-III, and PDMS tests, all available test scores were incorporated in the analysis.

### Statistical Analysis

Continuous variables are presented as median (interquartile range), and categorical variables are described using frequencies and percentages. Chi-square and Mann Whitney tests were used to compare binary and continuous characteristics between the patients from Outcome in TOF that were evaluated in the CKPD clinic (this report) and those that were not.

### Mixed Effects and Marginal Models to Identify Factors Associated with Neurodevelopment Outcomes

Longitudinal data analysis methods were used to test the association between each predictor (exposure) and ND test scores (outcome). Mixed effects linear regression (or marginal models for BINS) was run to address the correlation of test scores within each patient because the neurodevelopmental test data are both longitudinal (collected across visits) and clustered (subtests within each test). Given the small sample size, we could not implement multi-level models with more than two levels to take into account additional nesting such as the correlation of scores within the same visit and within the same subtest. A separate regression model was run for BINS, Bayley-III, and PDMS, and each predictor. All models were adjusted for the presence of genetic syndrome.

For the Bayley-III and PDMS, we used transformed scores to have a mean of 100 and standard deviation of 15. Higher scores indicate better performance. All available scores per patient across visits and subtests were used. Mixed effects linear regression models used fixed effects including the predictor of interest, type of subtest, and testing visit number. A random intercept was included for each patient to adjust the model for the variability in outcome across patients. The estimate (ß coefficient) and 95% confidence interval for each significant predictor are provided.

The BINS outcome is categorical (emerging/at risk vs. normal development), so a marginal model with a logit link and computed robust standard error estimates for clustering within patient was used. The model for the BINS provided the estimate for the effect of each predictor, adjusted for subtest, test administration, and genetic syndrome. Odds ratios (OR) and 95% confidence intervals are reported for the predictors of interest.

*Associations between Predictors and Referral for Early Intervention:* Referral for early intervention is a binary outcome; therefore, a logistic regression model was run for each predictor, controlling for genetic syndrome. Odds ratios and 95% confidence intervals are presented.

Because genetic syndromes were associated with multiple outcomes and some predictors, models were adjusted for genetic syndrome as a confounder. We were not able to adjust for other potential confounders due to sample size restrition.

### Sub-analysis to Test Relationships Between Tests

We calculated correlation coefficients to assess how screening tests performed in infancy were associated with tests performed in childhood. Point biserial correlations estimated the correlation between the first BINS examination (emerging/at risk scores vs. normal score) with first and second administration Bayley-III scores comparing the motor, cognitive, and language subtests. Spearman correlations were used to examine the correlation between motor subtest scores (fine and gross motor) PDMS and Bayley-III tests.

Analyses were run using SAS software version 9.4. All statistical tests were two sided with an alpha of 0.05 as the set level of significance.

## Results

### Description of the Patient Population

Of the 146 participants from Outcome in TOF, there were 49 patients who met inclusion criteria and are reported in this study. (Table 1) Most patients were male (67%) and white (57%). Median gestational age was 38 weeks (interquartile range (IQR) 37,39). There were 20 patients (41%) with a genetic diagnosis, of whom 5 (10%) had 22q11 0.2 deletion syndrome and 6 (12%) had trisomy 21. Examples of other genetic diagnoses included mosaic tetrasomy 8 (a variant of unknown significance on the Noonan syndrome panel), and FLT4 mutation. The median age at TOF repair was 90 days (IQR 22,133). The median oxygen saturation was 92% at the time of TOF repair (IQR 81,96). Three patients had prior palliation, and ten (20.4%) underwent TOF repair in the neonatal period (≤ 30 days of age). The length of hospital stay following TOF repair was 7.5 days (IQR 6,12).

Compared to patients in Outcome in TOF that were not part of this analysis, this group had a higher prevalence of genetic syndromes (41% vs. 20% respectively, p = 0.01) and was younger at the time of TOF repair (median: 90 days vs. 111 days, p = 0.02), with a higher rate of neonatal TOF repair (20% vs. 10%, p = 0.004). In terms of neighborhood characteristics, in this group, there was a higher prevalence of high school dropouts (8% vs 6%, p = 0.01) and vacant households (10% vs 7%, p = 0.02). (Table S2).

### Results of Neurodevelopmental Tests

Of the 49 patients, 37 (76%) underwent baseline screening evaluation with the BINS at a median age of 4.5 months (IQR 3–6). Thirty-eight (78%) patients had the first PDMS administered at a median age of 4 months (IQR 3–6). Twenty-nine (59%) patients had the first Bayley-III administered at a median age of 13 months (IQR 12–17.5). (Table 2) There were 22 patients who had at least one administration of the BINS and Bayley-III and 24 patients with at least one administration of the PDMS and Bayley-III. Twelve patients did not undergo initial BINS testing due to initial age of evaluation, and 12 patients were lost to follow up beyond the first evaluation in infancy. There were no differences in neighborhood-level characteristics between patients with follow-up in the CKDP and those that were lost to follow up.

**Table 2:** Neurodevelopment test results

BINS					
First administration of BINS (*n*=37 patients) number of emerging or at risk scores (*n*; %)			Second administration of BINS (*n*=13 patients) number of emerging or at risk scores (*n*; %)		
Age (months), median (IQR)^2^	4.5	(3,6)	Age (months), median (IQR)	8	(7,9)
Expressive language	9	24%	Expressive language	2	15%
Receptive language	13	35%	Receptive language	3	23%
Cognitive	11	30%	Cognitive	3	23%
Fine motor	11	34%	Fine motor	1	8%
Gross motor	16	43%	Gross motor	4	31%

Bayley-III					
First administration of Bayley-III (*n*=29 patients)			Second administration of Bayley-III (*n*=28 patients)		
Age (months), median (IQR)	13	(12, 17.5)	Age (months), median (IQR)	25	(24,26)
Language: median (IQR)	97	(79, 106)	Language: median (IQR)*	89	(75.5,98.5)
Scores <85 (*n*; %)	9	31%	Scores <85 (*n*; %)	10	36%
Scores <70 (*n*; %)	4	14%	Scores <70 (*n*; %)	4	14%
Cognitive: median (IQR)	95	(85, 100)	Cognitive: median (IQR)*	90	(80,95)
Scores <85 (*n*; %)	6	21%	Scores <85 (*n*; %)	7	26%
Scores <70 (*n*; %)	0	0%	Scores <70 (*n*; %)	4	15%
Motor: median (IQR)	91	(76, 100)	Motor: median (IQR)	91	(85,94)
Scores <85 (n; %)	12	43%	Scores <85 (*n*; %)	6	24%
Scores <70 (n; %)	5	18%	Scores <70 (*n*; %)	3	12%

PDMS					
First administration of PDMS (*n*=37 patients)			Second administration of PDMS (*n*=17 patients)		
Age (months), median (IQR)	4	(3,6)	Age (months), median (IQR)	8	(7,9)
Fine motor: median (IQR)	100	(97,103)	Fine motor: median (IQR)	97	(91,97)
Scores <85 (n; %)	3	8%	Scores <85 (n; %)	2	12%
Scores <70 (n; %)	0	0%	Scores <70 (n; %)	1	6%
Gross motor: median (IQR)	95	(89,100)	Gross motor: median (IQR)	96	(85,100)
Scores <85 (n; %)	4	11%	Scores <85 (n; %)	4	24%
Scores <70 (n; %)	0	0%	Scores <70 (n; %)	1	6%

*Significant changes in scores from first test administration (*p* < 0.05)

There were deficits in at least one domain on the BINS in 43% of patients. Gross motor deficits were the most common (*n* = 16), followed by deficits in receptive language, cognitive, and fine motor skills, which were seen in one-third of patients.

The median composite scores on the PDMS and Bayley-III tests were lower than the normative population’s expected scores. (Table 2) Test scores were particularly lower in patients with trisomy 21 and 22q11 deletion syndrome, as compared to those with either no genetic syndrome or with other genetic defects of unclear significance.

### Factors Associated with Neurodevelopment Outcomes

Several neighborhood-level factors related to poverty were associated with greater odds of abnormal neurodevelopment, most notably on the early screening BINS evaluation, including a higher block group percent unemployment rate, lower median household income, and a larger proportion of individuals below 100% of the poverty line. (Table 3).

**Table 3:** Factors associated with neurodevelopment outcomes

Factors	BINS (emerging or at risk)		
	Odds ratio	95% CI	*p*-value
Socioeconomic factors			
Median household income*	0.29	–0.11, 0.78	0.015
Percent unemployment*	2.7	1.29, 5.67	0.0085
Low-income persons (< 100% poverty level)*	3.3	1.49, 7.35	0.0034
Medical history			
Prostaglandin use	0.032	0.0020, 0.51	0.015
Oxygen saturation at time of repair	0.94	0.88, 1.00	0.067
Neonatal repair	0.044	0.0022, 0.87	0.04
Discharged home prior to full TOF repair	0.098	0.014, 0.66	0.017
Anatomy			
Pulmonary valve stenosis	0.18	0.035, 0.92	0.039
Abnormal Coronary	3.28	1.19, 9.03	0.022
Extracardiac Malformation	6.25	0.95, 41.26	0.057
AP collaterals	7.6	1.10, 52.63	0.04
Tetralogy repair surgery and post-operative course			
Total CPB time, minutes	1.01	1, 1.03	0.051
Use of DHCA^**^	0.32	0.11, 0.95	0.04
Cardiac complications	1.96	1.08, 3.55	0.027
Non-cardiac complications	7.18	2.19, 23.54	0.0011

	PDMS		
	ß Coefficient	95% CI	*p*-value
Medical history			
Birth weight	7.14	2.15, 12.13	0.0055
Oxygen saturation at time of repair	0.39	− 0.025, 0.81	0.065
Anatomy			
AV Canal	− 26.8	− 42.37, − 11.22	0.0009
Tetralogy repair surgery and post-operative course			
Number of CPB runs	− 8.41	− 16.10, − 0.71	0.033
Total CPB time	− 0.074	− 0.15, − 0.00040	0.049
Non-cardiac complications^***^	− 4.69	− 8.11, − 1.28	0.0075

	Bayley-III		
	ß Coefficient	95% CI	*p*-value
Medical history			
Oxygen saturation at time of TOF repair	0.47	− 0.0019, 0.93	0.051
Tetralogy repair surgery and post-operative course			
Cross-clamp duration	− 0.12	− 0.25, 0.011	0.072
CPB duration	− 0.09	− 0.19, 0.0081	0.072
Non-cardiac complications	− 4.35	− 8.33, − 0.37	0.032

^*^Estimates presented for a 1 standard deviation unit change in predictor

^**^DHCA: Deep hypothermic circulatory arrest

^***^Non-cardiac complications included: seizure, pneumothorax, and pleural effusion

Complex cardiac anatomy (presence of aortopulmonary collaterals and TOF with atrioventricular canal), longer duration of cardiopulmonary bypass, and greater number of cardiac and non-cardiac post-operative complications were associated with worse neurodevelopment outcome across the various tests administered. (Table 3) Duration of hospitalization after TOF repair was not associated with neurodevelopment outcomes. Additional factors such as use of prostaglandin therapy prior to TOF repair and neonatal TOF repair were associated with lower odds of abnormal neurodevelopment on early screening with the BINS. (Table 3).

Non-significant predictors are available in Table S2.

### Factors Associated with Referral to Early Intervention

In total, 19 patients (39%) were referred to early intervention. Factors associated with greater odds of referral included TOF repair in the neonatal period, history of prostaglandin use prior to TOF repair, and use of deep hypothermic circulatory arrest during TOF repair. Neighborhood’s prevalence of high school dropouts and patient’s discharge home after birth (to be readmitted later for TOF repair) were associated with lower odds of referral (Table 4).

**Table 4:** Factors associated with referral to Early Intervention

	Referral to early intervention		
Factors	Odds ratio	95% CI	*p*-value
Demographic			
Male	0.12	0.024,0.64	0.012
Socioeconomic factors			
Neighborhood median household income*	2.74	1.10, 6.85	0.031
Neighborhood % high school dropout*	0.11	0.059, 0.85	0.028
Medical history			
Prostaglandin use prior to TOF repair*	3.05	1.26, 7.37	0.014
Discharged home after birth	0.063	0.009,0.46	0.0063
Neonatal repair (< = 30 days of life)*	0.42	1.18, 5.82	0.019
Tetralogy repair surgery			
DHCA use during TOF repair**	2.25	1.07, 4.73	0.032
Lowest temperature on CPB	0.87	0.78, 0.98	0.020

*Estimates presented for a 1 standard deviation unit increase in predictor

**DHCA: Deep Hypothermic Circulatory Arrest

### Sub-analysis of Correlations Between BINS and PDMS with Bayley-III Tests

The first administration of the BINS evaluation was used, and each domain was compared separately: language, fine motor, gross motor, expressive language, and receptive language. Patients were classified as at risk/emerging vs. normal development for each domain. (Table 5) There were moderate and strong correlations between abnormal initial BINS evaluation and lower Bayley-III scores across all subtests. (Table 6) Similarly, there were moderate correlations between the initial PDMS and subsequent Bayley-III (Table 7).

**Table 5:** Summary of initial BINS and subsequent Bayley-III scores

First administration of BINS	Subsequent test	*N*	Median	IQR
Fine motor subtest				
Normal development (*n* = 21 patients)	1st Bayley Motor	13	102.5	93.6–111.5
	2nd Bayley Motor	10	107.0	99.5–111.5
Emerging/at risk development (*n* = 11 patients)	1st Bayley Motor	5	80.6	72.7–87.6
	2nd Bayley Motor	4	93.6	72.7–104
Gross motor subtest				
Normal development (*n* = 16 patients)	1st Bayley Motor	9	111.5	99.5–114.5
	2nd Bayley Motor	8	110.0	102.5–111.5
Emerging/at risk development (*n* = 16 patients)	1st Bayley Motor	9	80.6	72.7–87.6
	2nd Bayley Motor	6	93.6	66.7–105.5
Cognitive				
Normal development (*n* = 26 patients)	1st Bayley Cognitive	17	106.5	106.5–116.4
	2nd Bayley Cognitive	16	101.5	96.6–106.5
Emerging/at risk development (*n* = 11 patients)	1st Bayley Cognitive	5	91.6	86.6–96.6
	2nd Bayley Cognitive	4	89.1	76.7–101.5
Expressive language				
Normal development (*n* = 28 patients)	1st Bayley Language	15	111.5	100.5–118.4
	2nd Bayley Language	15	100.5	88.6–108.5
Emerging/at risk development (*n* = 9 patients)	1st Bayley Language	7	88.6	70.7–114.5
	2nd Bayley Language	6	90.1	67.7–102.5
Receptive language				
Normal development(*n* = 24 patients)	1st Bayley Language	15	111.5	105.5–126.4
	2nd Bayley Language	15	102.5	88.6–111.5
Emerging/at risk development (*n* = 13 patients)	1st Bayley Language	7	88.6	70.7–111.5
	2nd Bayley Language	6	88.6	67.7–100.5

**Table 6:** Correlations between First BINS administration and subsequent Bayley-III

	1st Administration Bayley-III-related construct	2nd Administration Bayley-III-related construct
Emerging/at risk BINS fine motor development	− 0.58	− 0.42
Emerging/at risk BINS gross motor development	− 0.77	− 0.61
Emerging/at risk BINS expressive language development	− 0.37	− 0.39
Emerging/at risk BINS receptive language development	− 0.59	− 0.40
Emerging/at risk BINS cognitive development	− 0.57	− 0.49

^*^Correlations are point biserial correlations

**Table 7:** Correlations between first PDMS administration and subsequent Bayley-III using motor subscale scores

	1st Administration Bayley-III Motor Subtest	*p*-value	2nd Administration Bayley-III Motor Subtest	*p*-value
1st Administration PDMS Fine Motor Subtest	0.4	0.052	0.67	0.002
1^st^ Administration PDMS Gross Motor Subtest	0.55	0.01	0.64	0.004

## Discussion

Neurodevelopment has been understudied in infants after TOF repair. We sought to fill this knowledge gap and investigated patients operated for TOF that were evaluated in a specialized neurodevelopment program. We provide a preliminary analysis of the neurodevelopmental status of infants and children with TOF, and report on factors associated with neurodevelopmental outcomes. Our main findings include significant deficits identified in the screening tests, significant associations between socioeconomic factors and neurodevelopment, and in sub-analysis, significant correlations between early screening tests with the later-performed, more definitive Bayley-III evaluation.

We found a significant prevalence of abnormal neurodevelopment scores. These results are similar to Gaynor et al., who demonstrated abnormal neurodevelopment in patients reaching school age with various congenital heart defects, including TOF, using the Bayley-III test [21]. To our knowledge, our study is the first to demonstrate significant deficits detected in infancy using the BINS. Other studies report the use of this screening test among patients with low birth weight, those born prematurely, and those suffering from other neonatal complications [11, 12]. In these high-risk groups, deficits in the BINS predict neurodevelopmental deficits later in childhood [22, 23]. The sub-analysis in our study demonstrates that abnormal BINS was associated with lower scores in the Bayley-III performed after 1 year of age. To our knowledge, there are no other reports of use of the early BINS screener in patients with CHD in general, and our results suggest that the BINS could be of use for patients with TOF and possible other CHD.

Studies have reported that the PDMS detects deficits in motor functioning in 42% of young children with repaired CHD; however, the utility of early administration of the PDMS during infancy has not been reported [24]. The PDMS performed in neonates and infants might suggest risk of future fine and gross motor deficits, as suggested by our sub-analysis. Thus, the PDMS could be considered to indicate the need for early intervention therapy. Our findings emphasize the importance neurodevelopment testing in patients with TOF starting in infancy, similarly to what has become a standard of care for patients with transposition of the great arteries and hypoplastic left heart syndrome [5, 7, 21].

We report various socioeconomic factors associated with neurodevelopment, in particular with the early BINS and PDMS tests. Social and economic drivers have a known influence on multiple facets of CHD outcomes from birth through childhood [13, 25]. Lower socioeconomic status is also known to affect neurodevelopment in all children in multiple domains, including global measures of cognition such as IQ and academic achievement. The impact of socioeconomic status is multi-factorial, beginning prenatally, and is closely intertwined with factors such as maternal comorbidities, toxic stress, and environmental exposures, as well as food insecurity, potentially impacting illness courses, and concurrently, neurodevelopment [13, 26]. Further, studies in patients with transposition of the great arteries and hypoplastic left heart syndrome demonstrated that lower socioeconomic status was associated with greater odds of hospital readmissions, heart transplant, and death [14, 15]. Therefore, it is conceivable that low socioeconomic status confers additional risk of abnormal neurodevelopment in patients with TOF as well. Studies have demonstrated the association of maternal education with neurodevelopment in children with CHD [7, 27]. However, studies focused on social determinants of health in TOF are not available, in particular using novel geo-coding techniques.

Similarly to the study by Gaynor et al., we found that greater birth weight was associated with better neurodevelopment scores [6]. This association is likely due to the fact that fetuses with CHDs benefit from intra-uterine growth, including a possible beneficial effect of growth on brain maturation [28–30]. Therefore, gestation offers an opportunity to optimize fetal growth with adequate nutrition and care of maternal comorbidities, which could also be related to socioeconomic deprivation [26].

Finally, we demonstrate that factors representing disease complexity were associated with worse neurodevelopment. Thus, the interplay between disease complexity and neurodevelopment is also present in TOF, similarly to patients with other severe CHDs, including single ventricle heart disease and transposition of the great arteries [5, 7, 21].

## Limitations

We acknowledge limitations to this retrospective study. Our analyses were limited by the number of patients that presented for evaluation in the CKDP. The analysis of patients seen as part of clinical care resulted in loss to follow up, despite the protocol established by the CKDP clinic, which suggests yearly follow-up for all patients. We considered the possibility of referral bias to CKDP; however, the group that was evaluated was overall comparable to patients from Outcome in TOF that were not evaluated in the CKDP. Minor differences in socioeconomic indices were seen, which did not appear to be clinically relevant; thus, the possibility of referral bias based on socioeconomic status is less likely. Patients with genetic syndromes and extracardiac malformations were more likely to be evaluated, as expected, given that these patients are at a greater risk of abnormal neurodevelopment. The small sample size precluded inclusion of additional confounders in the models. Finally, this study was conducted in a large tertiary care center with a neurodevelopmental clinic focusing on children with congenital heart disease, thus, limiting the generalizability of our results. The CKDP has a well-trained staff available to administer testing to young infants with potentially limited endurance to tolerate prolonged evaluations, which might not be available at other centers.

## Conclusions

This preliminary report suggests that early testing with BINS and PDMS could be of clinical utility to screen patients with TOF and possibly other CHD, allowing for early implementation of a “medical home” comprising rigorous surveillance and therapies. Associations between the early tests and outcomes on Bayley-III further support this recommendation. Socioeconomic factors and disease complexity are associated with neurodevelopment and should be taken into account in the risk stratification and follow-up of these patients. These findings based on clinically indicated evaluations need validation in a larger prospective study.

## Supplementary Information

Below is the link to the electronic supplementary material.

Supplementary file1 (docx 22 KB)

## Abbreviations

TOF: Tetralogy of Fallot
CHD: Congenital heart disease
ND: Neurodevelopment/neurodevelopmental
CKDP: Cardiac Kids Developmental Follow-up Program
BINS: Bayley Infant Neurodevelopmental Screener
Bayley-III: Bayley Scales of Infant and Toddler Development, Third edition
PDMS: Peabody Developmental Motor Scale
CPB: Cardiopulmonary bypass
