# Supplementary material for: Early Evaluation and the Effect of Socioeconomic Factors on Neurodevelopment in Infants with Tetralogy of Fallot
Source: Pediatr Cardiol. 2021 Feb 3;42(3):643–53. doi: 10.1007/s00246-020-02525-6 (PMC7990815; doi:10.1007/s00246-020-02525-6)
Supplement: Supplementary file 1 — Supplementary file1 (docx 22 KB) [file 246_2020_2525_MOESM1_ESM.docx]

**Supplementary Materials**

**Table S1. Description of neurodevelopment tests administered**

| **Test** | **Age administered in study** | **Measures** | **Scores** |
| --- | --- | --- | --- |
| **BINS** | 3 -12 months | - Expressive and receptive language - Cognition - Fine and gross motor skill | - Normal  - Emerging - At risk |
| **PDMS** | 0-5 years | - Fine and gross motor skills | - Population mean: 100 - Standard deviation: 15 |
| **Bayley-III** | 12 months - 3 years | - Expressive and receptive language - Cognition - Fine and gross motor skills | - Population mean: 100 - Standard deviation: 15 |

**Table S2. Comparisons between patients in this analysis and those from Outcome in TOF not evaluated in the CKDP**

| **Demographics** | **Analyzed in this report (n=49)** | | **Outcome in TOF not seen in CKDP (n=146)** | |  |
| --- | --- | --- | --- | --- | --- |
|  | N | Percentage | N | Percentage | P-value |
| Male | 33 | 67% | 86 | 59% | 0.2 |
| Race |  |  |  |  | 0.2 |
| Non-Hispanic Caucasian | 28 | 57% | 113 | 77% |  |
| Hispanic | 10 | 20% | 12 | 8% |  |
| Non-Hispanic Black | 8 | 16% | 12 | 8% |  |
| Non-Hispanic Other | 2 | 4% | 9 | 6% |  |
| Primary caregiver education |  |  |  |  | 0.03 |
| Less than 12 years | 1 | 2% | 3 | 2% |  |
| 13-15 years | 18 | 37% | 29 | 20% |  |
| Greater than 16 years | 12 | 24% | 34 | 23% |  |
| Unknown | 18 | 37% | 74 | 51% |  |
|  |  |  |  |  |  |
| **Neighborhood Socioeconomic Factors** |  |  |  |  |  |
|  | Median | IQR | Median | IQR | P-value |
| Social Disadvantage Index (Z-Score) | -0.07 | (-0.69, 0.48) | -0.34 | (-0.88, 0.54) | 0.16 |
| Median Age | 36.85 | (33.8, 41.65) | 40.4 | (33.80, 44.90) | 0.0997 |
| Median Household Income | 69703 | (51731, 86807) | 75213 | (49236,100083.5) | 0.38 |
| Non-Hispanic African American | 6% | (1%, 23%) | 4% | (1%, 14%) | 0.44 |
| Households receiving Public Assistance | 2% | (0%, 5%) | 1% | (0%, 4%) | 0.095 |
| Female-Headed Households with Children under 18 | 8% | (3%, 12%) | 6% | (2%, 12%) | 0.26 |
| Hispanic | 7% | (3%, 15%) | 5% | (2%, 12%) | 0.083 |
| High School Dropouts | 8% | (6%, 16%) | 6% | (3%, 12%) | 0.0083 |
| Householders who moved into unit within the last 3 years | 4% | (3%, 13%) | 6% | (3%, 12%) | 0.94 |
| Without a vehicle | 4% | (2%, 13%) | 4% | (1%, 11%) | 0.17 |
| Households with > 1 person per room | 0% | (0%, 3%) | 0% | (0%, 3%) | 0.58 |
| Renter-Occupied Houses | 27% | (18%, 49%) | 25% | (11%, 49%) | 0.57 |
| Overall Unemployment | 5% | (3%, 9%) | 5% | (3%, 8%) | 0.42 |
| Vacant | 10% | (5%, 14%) | 7% | (2%, 12%) | 0.023 |
| Non-Hispanic White | 74% | (45%, 86%) | 77% | (53%, 90%) | 0.27 |
| Population Density (per square mile) | 3251.38 | (1070.82, 10760.49) | 3799.5 | (1554.39, 7818.82) | 0.83 |
| Low Income Persons (100-149% Poverty Level) | 4% | (1%, 11%) | 5% | (2%, 11%) | 0.96 |
| Low Income Persons (<100% poverty level) | 9% | (4%, 20%) | 6% | (2%, 15%) | 0.084 |
|  |  |  |  |  |  |
| **Medical History** |  |  |  |  |  |
|  | Median | IQR | Median | IQR | P-value |
| Gestational age (weeks) | 38 | (37,39) | 38 | (37,39) | 1 |
| Birth weight (kg) | 3.03 | (2.58, 3.41) | 2.97 | (2.51,3.49) | 0.3 |
| Head circumference at birth (percentile) | 18 | (8,31) |  |  |  |
|  | N | Percentage | N | Percentage | P-value |
| Extra cardiac Malformation | 31 | 63% | 47 | 41% | 0.0075 |
| Genetics diagnosis | 20 | 41% | 23 | 20% | 0.005 |
| 22q11 deletion | 5 | 10% | 10 | 9% | 0.77 |
| Trisomy 21 | 6 | 12% | 5 | 4% | 0.068 |
| Cardiac anatomy |  |  |  |  |  |
| Pulmonary valve anatomy: |  |  |  |  | 0.2 |
| Stenosis | 39 | 80% | 124 | 85% |  |
| Atresia/ Absent valve leaflets | 10 | 20% | 22 | 15% |  |
| TOF atrioventricular canal | 2 | 4% | 6 | 4% | 1 |
| Continuous pulmonary arteries | 46 | 94% | 138 | 93% | 0.8 |
| AP collaterals | 7 | 14% | 10 | 7% | 0.1 |
| Right aortic arch | 16 | 33% | 41 | 28% | 0.7 |
| Maternal-fetal environment |  |  |  |  |  |
| Umbilical artery pulsatility index, Median (IQR) | 1.11 | (0.97,1.3) |  |  |  |
| Smoking | 14 | 29% |  |  |  |
| Hypertension | 3 | 6% |  |  |  |
| Diabetes | 3 | 6% |  |  |  |
| Auto-immune disease | 0 | 0% |  |  |  |
| Maternal congenital heart disease (CHD) | 0 | 0% |  |  |  |
| Maternal Family History of CHD | 4 | 8% |  |  |  |
| Maternal Previous Child with CHD | 1 | 2% |  |  |  |
| Maternal Genetic Syndrome | 1 | 2% |  |  |  |
|  |  |  |  |  |  |
| **Surgical History** |  |  |  |  |  |
|  | N | Percentage | N | Percentage | P-value |
| Palliative surgery/intervention | 3 | 7% | 25 | 17% | 0.4 |
| Neonatal repair | 10 | 20% | 15 | 10% | 0.004 |
|  | Median | IQR | Median | IQR | P-value |
| Age at TOF repair operation (days) | 90 | (20, 133) | 111 | (79, 165) | 0.02 |
| Oxygen Saturation at the time of surgery (%) | 92 | (81.5, 96.5) | 93 | (86,98) | 0.1 |
| Weight at the time of surgery (kg) | 3.4 | (2.6, 4.38) | 3.72 | (2.86, 5.18) | 0.16 |
| BSA at the time of surgery (m2) | 0.22 | (0.19, 0.26) | 0.23 | (0.20, 0.29) | 0.12 |
| Intra-operative |  |  |  |  |  |
| Number of cardiopulmonary bypass runs | 1 | (1,1) | 1 | (1,1) | 0.99 |
| Total cardiopulmonary bypass time (in minutes) | 67 | (39, 93) | 64 | (41,86) |  |
| Total aortic cross-clamp time (in minutes) | 43 | (23, 57) | 47 | (28,65) | 0.2 |
| Deep hypothermic circulatory arrest |  |  | 7 | 5% | 0.2 |
| Lowest pH on cardiopulmonary bypass | 7.33 | (7.29, 7.35) | 7.33 | (7.29,7.37) | 0.2 |
| Lowest esophageal/nasal temperature in during CPB | 35.7 | (33.4, 36) | 35.3 | (34,36) | 0.3 |
| Lowest hematocrit on cardiopulmonary bypass | 37 | (33, 37) | 36 | (29,37) | 0.5 |
|  |  |  |  |  |  |
| **Post-operative complications** |  |  |  |  |  |
|  | N | Percentage | N | Percentage | P-value |
| Catheterization | 12 | 24% | 16 | 14% | 0.094 |
| Arrhythmia requiring treatment | 11 | 22% | 13 | 11% | 0.061 |
| Extra-corporal membrane oxygenation | 4 | 8% | 7 | 6% | 0.73 |
| Pleural effusion requiring chest tube | 4 | 8% | 4 | 3% | 0.24 |
| Cardiac arrest and cardiopulmonary resuscitation | 3 | 6% | 5 | 4% | 0.7 |
| Pneumothorax requiring drainage | 2 | 4% | 2 | 2% | 0.58 |
| Pericardial effusion requiring pericardiocentesis | 1 | 2% | 0 | 0% | 0.3 |
| Seizure requiring treatment | 1 | 2% | 1 | 1% | 0.51 |
| Post-operative length of stay (days), Median (IQR) | 7.5 | (6,12) |  |  |  |
|  |  |  |  |  |  |
| **Neurodevelopmental Interventions** |  |  |  |  |  |
|  | N | Percentage |  |  |  |
| Referred to Early Intervention | 19 | 39% | Not available |  |  |
| Received therapy | 29 | 59% |  |  |  |
| Physical therapy | 20 | 41% |  |  |  |
| Occupational therapy | 18 | 37% |  |  |  |
| Speech therapy | 18 | 37% |  |  |  |
| Behavioral therapy | 1 | 2% |  |  |  |
